# Analyze the clinical effect of YAG laser combined with sodium hypochlorite in root canal treatment of pulpitis

**DOI:** 10.1002/iid3.975

**Published:** 2023-09-26

**Authors:** Qiaolin Lin, Zhixin Li, Mingming Liu

**Affiliations:** ^1^ Department of Stomatology Shijiazhuang Fourth Hospital Shijiazhuang China; ^2^ Laboratory Center The First Hospital of Hebei Medical University Shijiazhuang China

**Keywords:** Nd: YAG laser, periodontal index, pulpitis, root canal treatment, sodium hypochlorite

## Abstract

**Objective:**

To compare and analyze the clinical therapeutic effects of sodium hypochlorite combined with Nd: YAG laser and sodium hypochlorite alone for root canal disinfection in patients with pulpitis.

**Methods:**

Patients with pulpitis were divided into control group and observation group according to random number table method. Both groups received root canal treatment, while the control group received root canal irrigation with 1% sodium hypochlorite. The observation group was irrigated with 1% sodium hypochlorite combined with Nd: YAG laser. Periodontal index, inflammatory index, life quality score and bacterial infection clearance rate of the two groups were compared before and 3 months after treatment.

**Results:**

The total effective rate of the observation group was 95.35%, which was higher than that of the control group 79.07% (*p* < .05). After 3 months of treatment, the periodontal index and inflammation level of both groups decreased, and the observation group was lower than that in the control group (*p* < .05). The life quality score and infection clearance rate of observation group were significantly higher than control group (*p* < .05).

**Conclusion:**

Compared with root canal irrigation with 1% sodium hypochlorite alone, sodium hypochlorite combined with Nd: YAG laser for root canal disinfection can significantly improve the therapeutic effect, relieve inflammatory reaction, and decrease bacterial infection.

## Graphical abstract

Nd: YAG laser combined with 1% sodium hypochlorite for root canal irrigation in patients with pulpitis showed positive significance in improving periodontal index, relieving inflammation and improving quality of life.

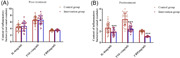

## 1 INTRODUCTION

Pulpitis is a common and frequently occurring disease in dental clinics. About half of patients with pulp diseases suffer from pulpitis. Pulpitis is a kind of dental pulp injury caused by bacterial infection, mechanical stimulation, chemical stimulation and other factors.^1^ Patients are prone to symptoms such as spontaneous parochial pain and temperature stimulation pain, which seriously affect patients' chewing function and daily diet. Generally, the damage caused by pulpitis is irreversible even after the trigger of inflammation is removed, mainly because the disease is usually accompanied by a loss of the ability of an organism to regenerate dental pulp.^2^ The blood system and nervous system in healthy dental pulp tissue can continuously supply nutrients to dental tissue and transport unwanted metabolic wastes through apical foramina. At the same time, in addition to dental pulp cells, there are many kinds of immune cells in healthy dental pulp tissue, such as T lymphocytes and macrophages, which can resist the invasion of foreign microorganisms and antigens.^3^, ^4^ However, the occurrence of pulpitis disrupted this stable environment. Therefore, once pulpitis is found, it is advisable to seek treatment in the stomatology department of the hospital immediately to avoid damage to the integrity of the teeth.

At present, the commonly used clinical treatment of pulpitis is root canal therapy after the infected root canals is completely removed.^5^ In most cases, root canal cleaning and shaping can be achieved by mechanical preparation.^6^ However, these instruments cannot reach all parts of the inner wall of the root canal, and infected pulp and residual dentin debris still exist in the inaccessible areas.^7^ In the process of root canal treatment, most microorganisms in the diseased tooth root canal can be removed by root canal preparation combined with irrigation solution, and subsequent drugs sealed in the root canal can completely kill the remaining microorganisms and prevent the recurrence of inflammation. The ideal irrigation solution should have the properties of antimicrobial activity, high permeability, dissolving necrotic tissue, so as to clear the lesions of root canal system of lesions, and be nontoxic to periapical tissue.^8^ Presently, the commonly used root canal irrigation solution includes sodium hypochlorite, chlorhexidine, chloramine T, strong acid electrolyte water, hydrogen peroxide solution, etc.^9^ As the most widely used flushing solution, sodium hypochlorite has a remarkable effect in root canal therapy and has become the “gold standard” of root canal flushing solution.^10^ Although sodium hypochlorite has excellent bactericidal effect, conventional irrigation with low concentration cannot completely remove bacteria from the dentin wall of root canal, which will undoubtedly reduce the success rate of root canal treatment. Meanwhile, traditional syringe needle irrigation is limited by complicated root canal anatomy and cannot be completely rinsed.^11^ Recently, with the application of laser in endodontic treatment, the combination of laser and flushing solution has been applied in clinics. Nd: YAG laser, with a wavelength of 1064 nm, can be strongly absorbed by water and hydroxyapatite in dentin to produce photothermal effects.^12^ Studies have shown that Nd: YAG laser combined with ordinary irrigation solution cannot only significantly improve the sterilization rate, but also effectively remove bacteria at different depths of dentin.

The objective of this study was to explore the effect of Nd: YAG laser combined with sodium hypochlorite on root canal irrigation in pulpitis patients by evaluating the changes of periodontal index and inflammatory factor levels before and after treatment, so as to find an effective root canal disinfection method in clinical practice.

## 2 MATERIALS AND METHODS

### 2.1 Study population and sample inclusion

Pulpitis patients were selected and divided into control group (*n* = 43) and observation group (*n* = 43) according to random number table method. Inclusion criteria: (1) The affected teeth have spontaneous pain, hot and cold stimulation pain or occlusion pain; (2) X‐ray examination confirmed lesion, alveolar bone resorption, and alveolar abscess; (3) The gums are red and swollen, and the teeth are loose by I and II degrees; (4) All patients had no treatment history of pulpitis. Exclusion criteria: (1) Patients with unclear root canal imaging and root canal blockage; (2) Patients with blood and immune system diseases; (3) Severely malnourished patients; (4) Patients with periapical disease; (5) Patients with a history of maxillofacial surgery; (6) Patients suffering from mental illness or disorder of consciousness; (7) Patients allergic to the drugs used in this study; (8) Pregnant and lactating women. All patients and their families in this study gave informed consent and this protocol was approved by the Ethics Committee of Shijiazhuang Fourth Hospital (Ethical number: 20200018).

The sample size was estimated using PASS 11.0 software based on a 5% false‐positive error rate (*α* = 0.05, bilateral) and 90% power (*β* = 0.1). A sample of at least 36 cases should be enrolled for each group. Assuming a dropout rate of about 20%, a total of 86 patients should have been recruited in this trial (43 per group).

### 2.2 Therapeutic method

Before treatment, all patients in both groups received routine oral cleaning and took antibiotics to control the inflammation. Both groups received one‐time root canal therapy. After the pulp was opened, the top of the pulp chamber was removed with a slow ball drill to prevent external contamination. After the root canal was cleaned, the root canal length was measured and the root canal was prepared with Dentsply Protaper system (Dentsply Maillefer, Baillagues, Switzerland). The control group was given sodium hypochlorite for root canal irrigation. The control group was given sodium hypochlorite (NaClO) for root canal irrigation, that is, 1% NaClO was washed first, then 0.9% sodium chloride solution was washed. The observation group was treated with Nd: YAG laser combined with 1% NaClO for root canal irrigation. Set the Nd: YAG laser (1064 nm, 15 Hz, 1.5 W) therapeutic instrument (France Guangtai Medical Company) to disinfection mode. The optical fiber was inserted 1 mm away from the root tip, and the root canal was irradiated and disinfected from bottom to top. Each root canal was irradiated four times in different directions for 5 s each time, with an interval of 5 s. After irradiation disinfection of each root canal, the exudation water was sucked out with absorbent paper, and the root canal was filled with hot gutta percha combined with i‐Rootsp paste under pressure.

### 2.3 Evaluation of periodontal index

Oral examination was performed before and 3 months after treatment to observe the periodontal index. Bleeding index (BI), with a score of 0−5. 0: no inflammation, no bleeding, healthy gums; 1 point: no bleeding but inflammatory changes in gingival color; 2 points: the gums are a little bleeding; 3 points: the bleeding spread along the gingival margin; 4 points: the amount of bleeding is large and overflows the gingival sulcus; 5 points: massive bleeding and automatic bleeding. Plaque index (PLI), with a score of 0−5. 0: aseptic plaque on the patient's tooth surface; 1 point: there is a small amount of plaque on the gingival margin; 2 points: there are continuous plaque in the tooth neck, and the width of plaques is less than 1 mm; 3 points: there are many dental plaques, and the coverage area attached to the tooth neck is more than 1 mm and less than 1/3 of the tooth area; 4 points: the attachment area of dental plaque accounts for 1/3 of the entire tooth surface; 5 points: plaque attachment area is more than 2/3 of the tooth surface. Gingival index, with a score of 0−3. 0: the gingiva is healthy as a whole, without any abnormality; 1 point: there is mild inflammation and edema in the gums; 2 points: the gums are red, with moderate inflammation and moderate edema and bleeding; 3 points: there is severe inflammation, obvious swelling and severe bleeding in the gums. The higher the score of the above periodontal index, the more serious the degree of tooth pain and bleeding.

### 2.4 Enzyme linked immunosorbent assay (ELISA)

The levels of TNF‐α, IL‐6 and CRP were determined by double‐antibody sandwich method according to the instructions of ELISA Kit. The sample was diluted 1:1 with 50 μL of specimen dilution and added to the reaction well, the remaining samples were added to each well, and then 50 μL of biotin antibody working solution was added, covered with membrane plate, gently mixed by shock, and incubated at 37°C for 30 min. Discard the liquid in the plate, add washing liquid to each hole, shake for 30 s, and repeat operation for 3 times. Then, chromogenic solution and the termination solution were added in turn, and the absorbance values of each hole at 450 nm were measured with enzyme‐labeled marker to calculate the sample concentration.

### 2.5 Quality of life assessment

The standardized questionnaire SF‐36 scale was used to assess patients' quality of life. The scale is used to assess subjects' functional status, emotional health, and physical health. Scores on each dimension of the SF‐36 scale ranges from 0 to 100, with higher scores indicating better quality of life.

### 2.6 Comparison of bacterial infection rates

Patients were examined for periodontal bacterial infection before and 3 months after treatment. Bacterial culture and drug sensitivity tests were carried out to calculate bacterial infection rate and infection clearance rate. Infection clearance rate = (Number of cleared cases/total cases) × 100%.

### 2.7 Therapeutic effect evaluation

The evaluation standard of curative effect can be used to evaluate the therapeutic effect according to the marked effect, effectiveness, and ineffectiveness. Effective: the clinical symptoms disappeared, the bite force and masticatory efficiency returned to normal, and the levels of various indicators returned to normal. Palliative: clinical symptoms were relieved, and the bite force, masticatory efficiency and various indexes were improved effectively. Useless: the clinical symptoms, bite force and masticatory efficiency have not been alleviated or improved. Total effective rate = (Marked effect + effectiveness)/total cases × 100.

### 2.8 Data analysis

SPSS19.0 software was used for data analysis and processing. Counting data is described in %. Kolmogorov‐Smirnov is applied to test whether the data confirms to the normal distribution, and the data fitting the normal distribution was described by mean ± standard deviation (SD). Levene method was used to check the uniformity of variance. Independent sample *t* test was used for intergroup comparison, and paired *t* test was used for intragroup comparison. *p* < .05 was considered statistically significant.

## 3 RESULTS

### 3.1 Comparison of baseline data

General data of the control group and the observation group are summarized in Table 1. There were no statistically significant differences between the two groups in gender, age, location of affected tooth and basic diseases (hypertension and diabetes), which was comparable (*p* > .05).

**Table 1:** Basic clinical information of the subjects.

Items	Control group (*n* = 43)	Observation group (*n* = 43)	T/*χ* ^2^	*p*
Age (years)	51.60 ± 11.29	50.98 ± 10.72	1.82	.07
Gender (*n*/%)			0.42	.52
Male	24 (55.82)	21 (48.84)		
female	19 (44.18)	22 (51.16)		
Hypertension (*n*/%)			0.66	.42
yes	7 (16.28)	10 (23.26)		
no	36 (83.72)	33 (76.74)		
Diabetes (*n*/%)			0.72	.40
yes	2 (4.65)	4 (9.30)		
no	41 (95.35)	39 (90.70)		
Location of affected tooth (*n*/%)			0.81	.37
Premolar	17 (39.53)	18 (41.86)		
Molar	26 (60.47)	25 (58.14)		

*Note*: Data presentation: mean ± standard deviation or *n*/%. *p* < .05 is considered a significant difference.

### 3.2 Comparison of periodontal index

**Figure 1:**
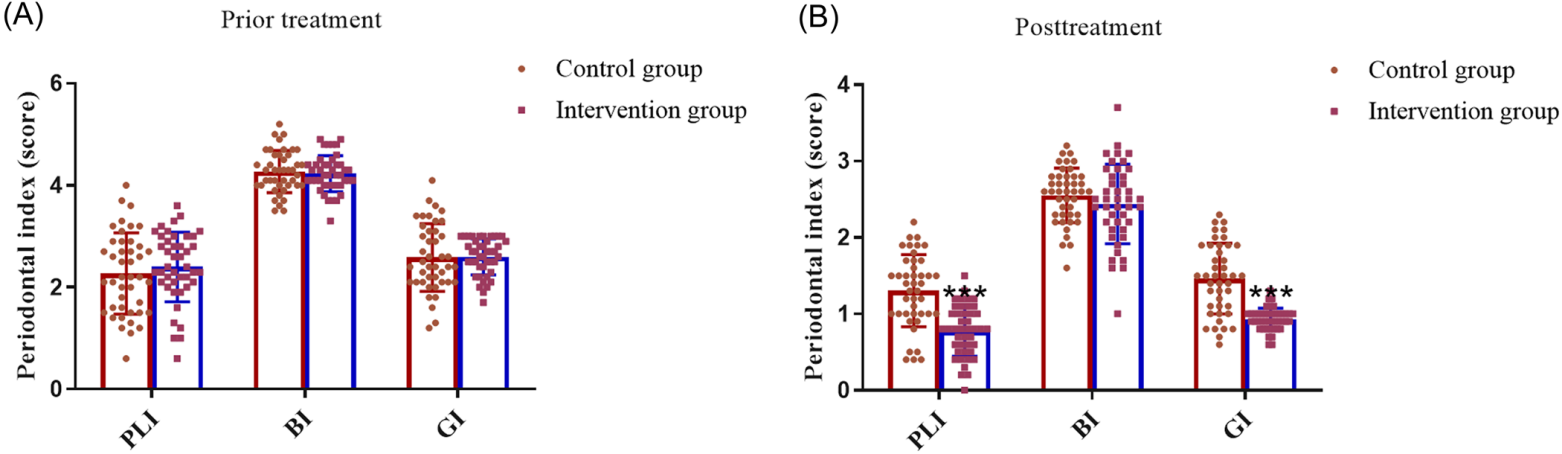
Detection of periodontal indicators. Assessment of PLI, BI and GI in control group (*n* = 43) and observation group (*n* = 43) before (A) and after treatment (B). ***p < .001 versus Control group using independent sample *t* test. BI, bleeding index; GI, gingival index; PLI, plaque index.

The results of intergroup comparison of periodontal index between the two groups are shown in Figure 1. Before treatment, the periodontal index including PLI, BI and GI scores of the control group and the observation group were nearly identical (Figure 1A, *p* > .05). After 3 months of treatment, except BI score, the scores of PLI and GI in the observation group were significantly lower than those in the control group (Figure 1B, *p* < .001). The intragroup comparison of periodontal index between the two groups was shown in Table 2. For both the control group and the observation group, the periodontal index score after treatment was significantly lower than that before treatment (Table 2, *p* < .01).

**Table 2:** Comparison of the periodontal clinical indexes between the two groups.

Items	Control group (*n* = 50)		*t*	*p*	Intervention group (*n* = 50)		*t*	*p*
	Before	After			Before	After		
PLI	2.27 ± 0.80	1.30 ± 0.47	8.17	<0.01	2.40 ± 0.68	0.78 ± 0.33	14.93	<.01
BI	4.27 ± 0.41	2.55 ± 0.36	23.25	<0.01	4.23 ± 0.35	2.44 ± 0.52	21.19	<.01
GI	2.59 ± 0.66	1.46 ± 0.46	8.94	<0.01	2.60 ± 0.36	0.93 ± 0.15	19.61	<.01

Abbreviations: BI, bleeding index; GI, gingival index; PLI, plaque index.

*Note*: Data presentation: mean ± standard deviation. *p* < .05 is considered a significant difference.

### 3.3 Comparison of inflammatory index before and after treatment

**Figure 2:**
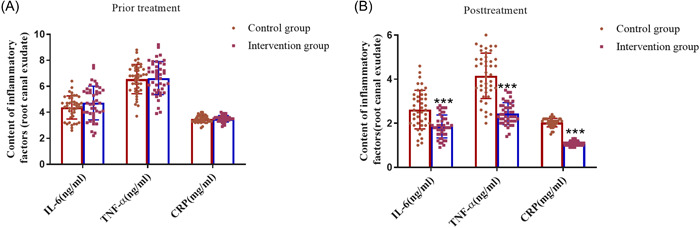
Detection of inflammatory indexes. Assessment of IL‐6, TNF‐α and CRP in control group (*n* = 43) and observation group (*n* = 43) before (A) and after treatment (B). ***p < .001 versus Control group using independent sample *t* test. CRP, C‐reactive protein; IL‐6, Interleukin‐6; TNF‐α, Tumor necrosis factor‐α.

Before treatment, there was no significant difference in IL‐6, TNF‐α and CRP between the control group and the observation group (Figure 2A, *p* > .05). After treatment, the levels of IL‐6, TNF‐α and CRP in the observation group were significantly lower than those in the control group (Figure 2B, *p* < .001). The results of intra‐group comparison of inflammatory indicators showed that the concentration of inflammatory indicators were significantly lower after treatment than before for both the observation group and the control group (Table 3, *p* < .01).

**Table 3:** Comparison of levels of inflammatory mediators in root canal exudate between the two groups.

Indicators	Control group (*n* = 43)		*t*	*p*	Observation group (*n* = 43)		*t*	*p*
	Before	After			Before	After		
IL‐6 (ng/mL)	4.37 ± 0.89	2.62 ± 0.87	8.01	<0.01	4.72 ± 1.30	1.86 ± 0.52	14.17	<.01
TNF‐α (ng/mL)	6.56 ± 1.13	4.15 ± 1.03	10.43	<0.01	6.63 ± 1.27	2.44 ± 0.50	19.38	<.01
CRP (mg/mL)	3.48 ± 0.27	2.03 ± 0.20	27.20	<0.01	3.50 ± 0.25	1.09 ± 0.10	56.54	<.01

*Note*: Data presentation: mean ± standard deviation. *p* < .05 is considered a significant difference.

### 3.4 Comparison of patients' quality of life before and after treatment

**Figure 3:**
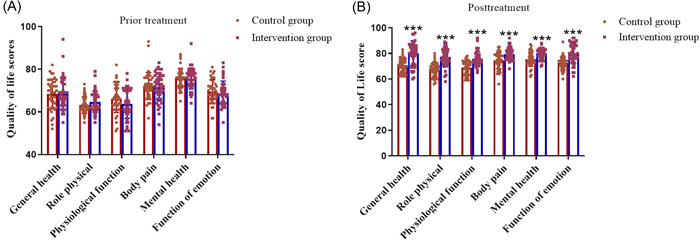
Quality of life assessment. Assessment of SF‐36 score in control group (*n* = 43) and observation group (*n* = 43) before (A) and after treatment (B). ***p < .001 versus Control group using independent sample *t* test.

Before treatment, there was no significant difference between the control group and the observation group in any item of the quality of life scores (Figure 3A, *p* > .05). After treatment, the scores of all aspects of life quality in the observation group were higher than those in the control group (Figure 3B, *p* < .001). The intragroup comparison of quality of life scores showed that after treatment, the scores of the control group were improved in all categories, but there were significant differences in physiological function and emotional function (Table 4, *p* < .05). However, for the observation group, compared with the control group, the scores in all categories increased after treatment (Table 4, *p* < .01).

**Table 4:** Comparison of quality of life scores between the two groups.

Items	Control group (*n* = 43)		*t*	*p*	Observation group (*n* = 43)		*t*	*p*
	Before	After			Before	After		
General health	68.40 ± 6.98	70.40 ± 5.43	−1.43	0.16	68.51 ± 7.31	80.16 ± 9.68	−5.40	<.01
Role physical	63.12 ± 3.87	67.30 ± 5.47	−3.93	<0.01	64.06 ± 5.06	77.26 ± 7.88	−8.72	<.01
Physiological function	66.16 ± 6.35	68.67 ± 5.44	−1.90	0.06	63.77 ± 6.83	75.79 ± 5.48	−9.05	<.01
Body pain	72.12 ± 6.36	74.47 ± 6.46	−1.98	0.05	71.65 ± 6.51	79.51 ± 4.87	−6.88	<.01
Mental health	75.79 ± 4.56	75.49 ± 5.47	0.29	0.77	75.65 ± 4.93	79.95 ± 3.97	−4.47	<.01
Function of emotion	69.58 ± 5.52	72.47 ± 5.08	−2.44	0.02	68.74 ± 5.69	79.77 ± 7.70	−6.89	<.01

*Note*: Data presentation: mean ± standard deviation. *p* < .05 is considered a significant difference.

### 3.5 Comparison of bacterial infection before and after treatment

The bacterial infection rate in both control and observation groups decreased significantly after treatment compared to before treatment (Table 5, *p* < .01). Additionally, it can be observed that the infection clearance rate in the observation group is significantly higher than that of the control group (Table 5, *p* < .01).

**Table 5:** Comparison of the periodontal clinical indexes between the two groups.

Items	Control group (*n* = 43)		*χ* ^2^	*p*	Observation group (*n* = 43)		*χ* ^2^	*p*
	Before	After			Before	After		
Bacterial infection rate	39 (90.70%)	9 (25.71%)	42.43	<.01	40 (93.02%)	2 (5.00%)	67.20	<.01
Infection clearance rate	30 (30%)				38 (52.6%)		10.90	<.01

*Note*: Data presentation: *n*/%. *p* < .05 is considered a significant difference.

### 3.6 Comparison of therapeutic efficacy between the two groups

After treatment, the total effective rate was 95.35% (41 cases) in the observation group, which was higher than that of the control group (79.07%, 34 cases) (Table 6, *p* < .05).

**Table 6:** Effect of different treatment methods on patients with acute pulpitis.

Items	Control group (*n* = 43)	Observation group (*n* = 43)	*χ* ^2^	*p*
Useless	9 (20.93%)	2 (4.65%)	5.11	.02
Palliative	10 (23.26%)	14 (32.56%)		
Effective	24 (55.81%)	27 (62.90%)		
Total effective rate	34 (79.07%)	41 (95.35%)		

*Note*: Data presentation: *n*/%. *p* < .05 is considered a significant difference.

## 4 DISCUSSION

At present, the main clinical treatment of pulpitis is root canal treatment, which can achieve good results.^13^ The root canal treatment of pulpitis requires the thorough removal of residual tissue and debris in the root canal system, as well as the effective elimination of bacteria and their complex product.^13^ However, the structure of the root canal system is relatively complex, and other areas except the principal root canal are irregular, which increases the difficulty of root canal treatment. During the preparation of the root canal, part of the wall of the root canal was difficult to be reached by instruments, so the removal of bacteria and their products was not complete. The residual bacteria after the filling of the root canal was the main cause of the failure of the root canal and the recurrence of periapical inflammation.^14^ Root canal flushing solution plays an important role in reducing bacteria in irregular areas of root canal, improving the success rate of root canal treatment, and promoting the healing of peri‐root tissue. In this study, it was found that Nd: YAG laser combined with 1% NaClO irrigation is better than 1% NaClO alone in reducing periodontal index, relieving inflammatory response, improving quality of life and reducing microbial infection.

For complicated root canal procedures, it is necessary to use effective antibacterial solution in the root canal. In the process of root canal therapy, the ideal root canal washing agent can effectively kill bacteria, dissolve necrotic tissue, lubricate the root canal, and remove the smear layer, which is the key to the success of root canal treatment.^15^, ^16^ As the most commonly used irrigating agent in root canal irrigation, NaClO not only has excellent antibacterial effect, but also has the advantages of high irrigation efficiency, low surface tension, and dissolving interstitial fluid and protein. In solution, NaClO dissociates into hypochlorous acid and hypochlorite, which kill bacteria by affecting their metabolism. Studies have shown that the bactericidal effect of NaClO was gradually enhanced with the increase of the concentration. When the concentration is higher than 0.05%, it will cause changes in cell vacuoles, but the antibacterial effect will be greatly weakened when the concentration is too low.^17^ Currently, the optimal concentration of NaClO for root canal irrigation has not been determined in clinic, and the common concentrations are 1%, 2.5%, and 5.25%.^18^ 5.25% NaClO will not only damage periapical tissues, but also cause chemical burns once it comes into contact with oral soft tissues.^19^ Considering the safety problem, 1% NaClO was selected in this study. At present, to improve the efficacy of irrigating agent, laser washing technology is gradually emerging in people's field of vision. Nd: YAG laser, as a solid‐state laser, can convert spectral energy into heat energy, remove most bacteria in root canal through thermal damage, and inhibit inflammatory reaction, which plays a good clinical role in the treatment of pulpitis.^20^, ^21^ Bergmans et al. showed that the dentin disc with a thickness of 1 mm was inoculated with actinomyces and streptococcus in the same proportion, and after three times of irradiation with 1.5w Nd: YAG laser, it was found that the morphology of bacteria gradually changed with the increase of irradiation times, and finally the bacteria were completely destroyed.^22^ The pulp tissue and periodontal tissue are anatomically communicating with each other, and there are mixed infections dominated by anaerobic bacteria in both the periodontal pocket and the infected pulp.^23^ The inflammation and immune response caused by them are similar. In this study, after laser‐assisted root canal cleaning, the periodontal index of patients, including PLI, BI, GI, and the levels of inflammatory indicators IL‐6, TNF‐α, and CRP, were all lower than those of the 1% NaClO group, which further indicating that the periodontal recovery and inflammation inhibition of patients after laser‐assisted root canal therapy were better than those in 1% NaClO alone. The above results also indicate that the periodontal problems of the patients have been alleviated correspondingly after the root canal treatment of pulpitis. Studies have shown that NaClO can only penetrate into dentin tubules about 130 μm, while some refractory pathogens, such as Enterococcus faecalis, can penetrate into the dentin at 300−1000 μm.^24^, ^25^ In this study, after treatment with Nd: YAG laser combined with 1% NaClO, the bacterial infection rate of patients was significantly reduced compared with 1% NaClO alone, indicating that laser irradiation played a positive role in killing bacteria in root canals and extinguishing their metabolites.

Due to the influence of time and other factors, the follow‐up time in this study was only 3 months after root canal therapy, and the long‐term effect was not clear. Current results also reflect only short‐term changes at 3 months after treatment. We intend to further expand the sample size and follow‐up time in future studies to explain the long‐term efficacy of this therapy. In summary, through the evaluation of this study, it was found that the effect of Nd: YAG laser combined with low concentration NaClO in root canal treatment was better than that of low concentration NaClO alone. Therefore, low concentration NaClO combined with laser may be a safe and convenient and reliable disinfection technique in root canal treatment.

## AUTHOR CONTRIBUTIONS

**Qiaolin Lin**: Conceptualization; data curation; funding acquisition; software; writing—original draft. **Zhixin Li**: Methodology; resources; supervision. **Mingming Liu**: Formal analysis; project administration; resources; writing—review and editing.

## CONFLICT OF INTEREST STATEMENT

The authors declare no conflict of interest.

## ETHICS STATEMENT

This protocol was approved by the Ethics Committee of Shijiazhuang Fourth Hospital. All patients and their families in this study gave informed consent.

## Data Availability

All data generated or analyzed during this study are included in this article. Further enquiries can be directed to the corresponding author.
